# Geometric Integration of Hybrid Correspondences for RGB-D Unidirectional Tracking

**DOI:** 10.3390/s18051385

**Published:** 2018-05-01

**Authors:** Shengjun Tang, Wu Chen, Weixi Wang, Xiaoming Li, Walid Darwish, Wenbin Li, Zhengdong Huang, Han Hu, Renzhong Guo

**Affiliations:** 1 Research Institute for Smart Cities, School of Architecture and Urban Planning, Shenzhen University, Shenzhen 518060, China; shengjuntang@szu.edu.cn (S.T.); measure@163.com (W.W.); xmingster@163.com (X.L.); zdhuang@szu.edu.cn (Z.H.); 2 Department of Land Surveying & Geo-Informatics, The Hong Kong Polytechnic University, Hung Hom 999077, Hong Kong, China; wu.chen@polyu.edu.hk (W.C.); w.darwish@connect.polyu.hk (W.D.); liwbiz@gmail.com (W.L.); huhan@whu.edu.cn (H.H.); 3 Faculty of Geosciences and Environmental Engineering of Southwest Jiaotong University, Chengdu 611756, China

**Keywords:** RGB-D, point cloud registration, bundle adjustment, feature matching, camera tracking

## Abstract

Traditionally, visual-based RGB-D SLAM systems only use correspondences with valid depth values for camera tracking, thus ignoring the regions without 3D information. Due to the strict limitation on measurement distance and view angle, such systems adopt only short-range constraints which may introduce larger drift errors during long-distance unidirectional tracking. In this paper, we propose a novel geometric integration method that makes use of both 2D and 3D correspondences for RGB-D tracking. Our method handles the problem by exploring visual features both when depth information is available and when it is unknown. The system comprises two parts: coarse pose tracking with 3D correspondences, and geometric integration with hybrid correspondences. First, the coarse pose tracking generates the initial camera pose using 3D correspondences with frame-by-frame registration. The initial camera poses are then used as inputs for the geometric integration model, along with 3D correspondences, 2D-3D correspondences and 2D correspondences identified from frame pairs. The initial 3D location of the correspondence is determined in two ways, from depth image and by using the initial poses to triangulate. The model improves the camera poses and decreases drift error during long-distance RGB-D tracking iteratively. Experiments were conducted using data sequences collected by commercial Structure Sensors. The results verify that the geometric integration of hybrid correspondences effectively decreases the drift error and improves mapping accuracy. Furthermore, the model enables a comparative and synergistic use of datasets, including both 2D and 3D features.

## 1. Introduction

Creating detailed 3D maps of indoor environments is critical for mobile robotics applications, including indoor navigation, localization and path planning. Simultaneous localization and mapping (SLAM) is key to reliable 3D maps, as it estimates the camera pose accurately, regardless of sensors [1]. Recently, the widespread availability of RGB-D sensors (such as Kinect and Structure Sensor devices) has led to rapid progress in indoor mapping. These tools have several advantages. They are low cost, lightweight and highly flexible and are capable of high-quality 3D perception [2,3]. However, despite these advantages, RGB-D’s depth measurements are limited by range (e.g., typically 0.5 to 3.5 m) [4,5]. As most RGB-D SLAM systems make use of information containing valid depth measurements, they ignore regions without 3D information. This is problematic for two reasons: first, it may result in an insufficient use of the information provided, second, it may introduce larger mapping errors in long-distance camera tracking or for scenes with greater depth variations [6], third the depth measurement limitation of RGB-D sensor could also cause “feature deficiency”, which means plenty of image key features without valid depth value may be ignored during camera tracking. Such a feature deficiency could cause problems throughout the spaces involved when modeling large-scale indoor environments, such as airports and churches. To remedy such difficulties, the correspondence from 2D image sequences could be used to provide extra information for the unmeasured areas. Consequently, features without valid depth information render the 2D constraint non-functional. However, this also provides new opportunities to use 2D correspondences to provide long-range constraints in the camera trajectory optimization process.

The geometric integration of hybrid correspondences for RGB-D tracking has rarely been investigated. In this paper, we propose a geometric integration model to improve the mapping accuracy of the RGB-D systems by globally minimizing the re-projection error of 2D and 3D correspondences across all frames. Our system consists of two components: an initial pose-tracking with 3D correspondences, and the geometric integration with hybrid correspondences. In the first section, we detail how the coarse pose tracking method generates the initial camera poses using 3D correspondences with frame-by-frame registration. These initial poses are used as inputs to the geometric integration model, which would to improve convergence during least square minimization. A geometric integration model is presented in detail for the integrated processing of 2D correspondences, 2D-3D correspondences, and 3D correspondences. In the following section, we also discuss the existing researches on the use of features in RGB-D SLAM. Section 3 describes the generic methodological structure of the proposed geometric integration of hybrid correspondences. Section 4 provides a comprehensive qualitative and quantitative evaluation of the system using datasets collected by calibrated Structure Sensor devices. Finally, some concluding remarks are presented and discussed in Section 5.

## 2. Related Work

In the past few years, various 3D dense mapping and SLAM pipelines that rely purely on RGB-D devices have been proposed. Rather than focusing on all visual SLAM systems, we review the research on RGB-D SLAM. The accuracy of the 3D maps produced by RGB-D devices is highly dependent on the accuracy of the frame registration. RGB-D SLAM systems can be categorized into two types based on the registration method: the dense style first proposed by [3] and the sparse style first proposed by [7]. 

In the first dense system, all of the depth data streamed from the Kinect sensor were fused into a single global volumetric model. For each depth frame, the camera pose was obtained by tracking the live depth frame to the global surface model using an ICP algorithm. However, the system was memory intensive and only worked well for mapping space with volumes of less than $7\;m^{3}$. Also, this system did not address the inevitable drift error that occurs during frame alignment when there is a long camera trajectory. Most of the following researchers have focused on extending the measurement range of KinectFusion by reducing its memory consumption. Whelan et al. extended the range of operation by using a rolling reconstruction volume and color fusion [8]. Zeng et al. used an octree-based structure on the GPU instead of an explicit voxel representation in KinectFusion [9]. They changed the update mechanism of reconstruction, which enabled the system to map 3D scenes eight times larger than the original KinectFusion. Both methods increased the likelihood of drift within the map, and neither provided any means of loop closure or global optimization. A hierarchical data structure proposed by [10] significantly reduced memory consumption during scene mapping, and Nießner et al. explored a new system for large-scale volumetric reconstruction based on a spatial hashing scheme [11], however these systems still lack drift correction. In addition, Henry et al. describe a multiple-volume representation to enable the creation of globally consistent maps of indoor environments [12]. However, this system cannot achieve real-time processing. Instead of tracking the camera pose with the accumulated model, Kahl et al. represented the depth information with signed distance functions and estimated the camera movement by minimizing the distance between them [13]. This method significantly reduced the computation cost relative to ICP-based methods such as KinectFusion. Keller et al. designed a new system to enhance the robustness of the dynamic scenes by using a point-based surfel representation, although this still produced huge drift errors [14]. Meilland and Comport proposed a new approach which combine the advantages of volumetric and key-frame based approaches [15]. This method fuses multiple reference images to create a single predicted frame. Then the fused frames can easily be transformed into a volumetric 3D model and used for registration. Zhou and Koltun present a dense scene reconstruction method with points of interest to preserves detailed geometry of objects [16]. The experiments results indicates that this method is able to produce more accurate camera trajectories than Extended KinectFusion or RGB-D SLAM. Stückler and Behnke proposed a method to find the spatial constraints between key frames by representing the RGB-D images with 3D multiresolution octree maps [17]. Whelan et al. presented a volumetric fusion-based SLAM system [18]. They represented the volumetric reconstruction data structure with a rolling cyclical buffer and used a non-rigid map deformation to improve surface reconstruction. However, this system did not work when reintegrated into the volumetric fusion frontend. Thomas and Sugimoto propose a two-stage framework to build in details and in real-time a large-scale 3D model based on the idea of structured 3D representation with planar patches [19]. 

Compared to dense RGB-D SLAM systems, a feature-based SLAM system uses few meaningful points for camera pose estimation, thus has a lower computational cost. In the early feature-based RGB-D SLAM system proposed by [20], the speeded up robust features (SURF) extracted from the color images were mapped to the depth image and the corresponding 3D points were used for camera pose estimation. Pose graph optimization was used for global consistency. Huang et al. developed a similar system that enabled 3D flight in a cluttered environment [21]. Based on previous research, Steinbrucker et al. integrated a linearization of the energy function to find the best rigid body motion between two frames, although without drift correction [22]. Henry et al. presented an RGB-D ICP algorithm for 3D mapping that combined visual feature matching with dense point cloud alignment [23]. This method overcome the limitations of tracking in areas that lack visual information. Endres et al. presented an evaluation scheme that investigated the accuracy, robustness, and processing time of three different kinds of feature descriptors [2]. In their system, they optimized the 3D pose graph using the g^2^o framework [24]. Kerl et al. derived a probabilistic formulation to estimate the camera motion [25,26]. Because the accuracy of the frame registration can be easily influenced by the random error of 3D correspondences, Khoshelham et al. proposed an epipolar search method based on the theoretical random error of the depth measurement, which used the depth error to weight the 3D correspondences during camera pose tracking [27]. Based on [27], Santos et al. and Vestena et al. presented an adaptive coarse-to-fine registration method for RGB-D mapping [7]. Each point used for registration was weighted based on the theoretical random error of the depth measurement. Steinbrucker et al. used a multiscale octree structure to represent a signed distance function [28]. This method allows a measurement volume of 45 m × 12 m × 3.4 m. Chow et al. presented a 3D terrestrial LiDAR system, which integrated with a MEMS IMU and two Microsoft Kinect sensors to map indoor urban environments. This system provides an effective means for mapping areas with texture less walls. However the systems still lack drift correction [29]. Mur-Artal and Tardos present a complete SLAM system called ORB-SLAM2 for monocular, stereo and RGB-D cameras [30], which includes map reuse, loop closing, and relocalization capabilities. However, the theoretical errors of depth measurements and the residual errors during frame registration are not considered in this system. 

The above discussion shows that most of the RGB-D SLAM constraints are related to either memory consumption or the lack of robust loop closure and global consistency. The use of region without valid depth has rarely been investigated in existing RGB-D SLAM systems. Hu et al. (2012) proposed a heuristic switching method, which can be used to choose between an RGB-D and a monocular visual method [31]. Zhang et al. used both 2D and 3D correspondences to recover relative poses. Initial depth is associated with features in two ways: from a depth map and by triangulation using estimated motion [32]. Ataer-Cansizoglu et al. utilized a similar method [6]. Based on the visual point features, they added the plane measurement to the bundle adjustment model. The bundle adjustment models in both systems optimize the camera trajectory by minimizing the square distance error in object space. The 3D coordinates of the correspondence are fixed and only the camera poses would be corrected during the optimization process. Although the weight of the correspondence is assigned according to depth type, the effectiveness of the algorithm is highly dependent on the estimated depth by triangulation. Tang et al. introduced an enhanced RGB-D mapping method to enlarge the measurement distance of RGB-D sensors [33]. The 3D scene generated from RGB sequences based on the structure from motion (SFM) method is used to supplement the 3D scene produced by depth images. This approach makes full use of 2D features to enlarge scene volume and enrich detail, but does not yield any improvement for camera poses. 

In the present study, we seek to build upon and improve these various methods by incorporating new considerations and constraints to enhance the integration of 2D and 3D information while simultaneously improving the tracking accuracy of RGB-D sensors. Our method can also handle these problems by exploring visual features whose depth is both available and unknown. Compared with previous work, this paper presents the following novel findings. First, 2D feature matches provided complementary constraints to 3D feature matches, which can improve the mapping accuracy and camera trajectory significantly, especially during unidirectional RGB-D tracking. Second, instead of fixing the depth of 3D correspondences, our strategy is to globally minimize the re-projection error of 2D and 3D correspondences across all frames. This has the advantage of taking into account the same visual features appearing in multiple frames, which can be used to adjust the estimated 3D locations of feature points and camera poses, making the optimization more robust to uncertain depth estimates. Third, the initial camera poses generated with frame-by-frame registration are used as inputs in the geometric integration model along with 3D correspondences, 2D-3D correspondences and 2D correspondences identified from frame pairs to improve overall convergence during the global optimization process.

## 3. Geometric Integration of Hybrid Correspondences

### 3.1. Overview of the Approach

The geometric integration model integrates hybrid correspondences according to a rigid mathematical method. Key points are extracted from the RGB-D frames and categorized as 2D and 3D feature points according to the depth validity of the features. Based on the feature matching method, the correspondences are then organized into three categories: 2D correspondences, 2D-3D correspondences and 3D correspondences. First, a camera tracking strategy involving 3D correspondences is used to obtain the initial camera pose while also considering the depth uncertainty. The ground coordinates of each 3D correspondence are averaged with the ground value of two corresponding 3D features. For the 2D-3D correspondences, the depth is associated with the correspondence from the key point with a valid depth value. For the 2D correspondences, the 3D coordinates are obtained by stereo triangulation based on the initial camera poses. Finally, the initial camera poses are used as inputs in the geometric integration model together with 3D correspondences, 2D-3D correspondence and 2D correspondences identified from frame pairs.

**Figure 1:**
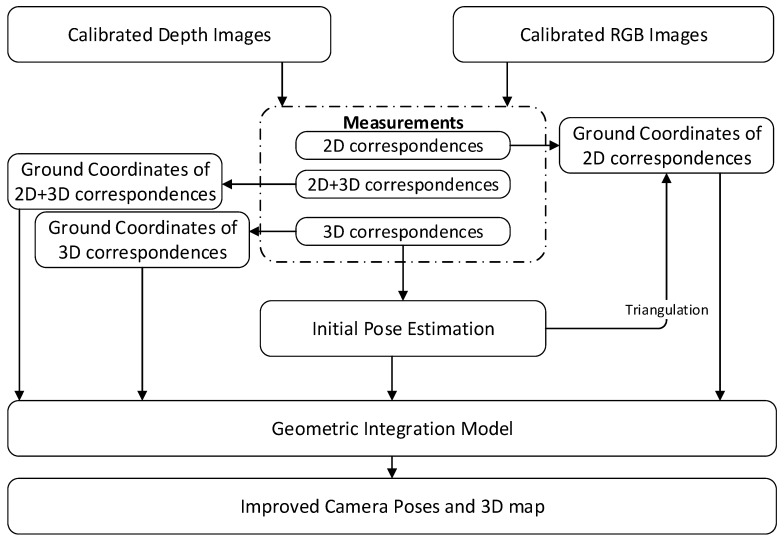
Framework of the geometric integration approach.

The framework of the geometric integration approach is shown in Figure 1. The camera poses and the 3D coordinates of 3D, 2D-3D and 2D correspondences are integrated with a re-projection error model. By globally minimizing the re-projection error of 2D, 2D-3D and 3D correspondences across all frames, the model ultimately improves the camera poses and mapping accuracy. It should be noted that, during the bundle adjustment processing, the poses of all keyframes and the 3D information of the feature points are optimized, except for the original keyframes that are fixed to eliminate the gauge freedom.

### 3.2. Preliminaries

This section provides mathematical preliminaries for the depth and RGB camera model. In the following mathematical expressions, matrixes and column vectors are denoted by bold letters, and scalars are written in italics.

#### 3.2.1. Depth Camera Projection Model

Depth camera projection model describes the relationship between image space and object space by using a pinhole camera model. With the interior parameters and the lens distortion of the depth camera, each pixel on the depth image can be projected to object space and the corresponding 3D coordinates can be obtained.

In the depth camera projection model, each pixel p is defined by its 2D pixel coordinates ${\left(x,y\right)}^{T}$, while the 3D point $P_{c}$ in the coordinate system of the current depth frame is described by three coordinates ${\left(X_{C},Y_{C},Z_{C}\right)}^{T}$. The depth camera model is given by:

(1) $$p=\frac{1}{d}K_{d}P_{c},$$

where $K_{d}=\left[\begin{array}{ccc} f_{dx} & 0 & c_{dx}\\ 0 & f_{dy} & c_{dy}\\ 0 & 0 & 1 \end{array}\right]$ and represents the interior matrix of the depth camera; $f_{dx}$ and $f_{dy}$ are the focal length of the depth camera; $c_{dx},\;c_{dy}$ are the principal point of the depth image, considering lens distortion; and d is the depth measurement of the specific pixel, which is equal to the z value of $P_{c}$. Given a pixel coordinates and its depth measurement, we can reconstruct the corresponding 3D point. 

#### 3.2.2. Pose Correlation between Depth and RGB Camera Model

In the initial pose tracking system, we compute the pose update by minimizing the discrepancies of the 3D correspondences from different frames. It means that the initial poses are obtained based on the depth camera model. As the camera poses are optimized by minimize the reprojection error on the image space, the pose correlation between depth and RGB camera model should be recovered and then used for poses optimization. The details are descripted as following.

The initial poses are related to the depth camera, which typically consists of a 3D rotation $R_{d}$ and a 3D translation $t_{d}$. It is convenient to transform the 3D structure from the camera coordinate system to a global coordinate system:

(2) $$P_{w}=R_{d}P_{c}+t_{d},$$

where the subscripts c and w denote the camera coordinate system and the global coordinates system, respectively, and where $P_{w}={\left(X_{w},Y_{w},Z_{w}\right)}^{T}$ is the homogeneous representation of a point in global coordinate system. By combining Equations (1) and (2), the pose update model can be rewritten as Equation (3):

(3) $$P_{w}=R_{d}*dK_{d}{}^{-1}p+t_{d},$$

However, unlike the initial camera tracking system, the geometric integration model optimizes the camera trajectory by minimizing the residual errors in the image space. The pinhole model is used and a scene view is formed by projecting 3D points into the image plane using a perspective transformation:

(4) $$zp=K_{rgb}\left(R_{rgb}P_{w}+t_{rgb}\right),$$

where $K_{rgb}=\left[\begin{array}{ccc} f_{rgbx} & 0 & c_{rgbx}\\ 0 & f_{rgby} & c_{rgby}\\ 0 & 0 & 1 \end{array}\right]$ and represents the interior matrix of the RGB camera; $f_{rgbx}$ and $f_{rgby}$ are the focal length of the RGB camera; and $c_{rgbx}$, $c_{rgby}$ are the principal point of the RGB image, considering lens distortion. The joint rotation-translation matrix $\left[R_{rgb},t_{rgb}\right]$ is called a matrix of the RGB camera’s extrinsic parameters.

Thus, the camera model problem of the geometric integration system now mainly consists of how to transform the initial camera pose $\left[R_{d},t_{d}\right]$ to the pinhole camera model used in the geometric integration system. By making a cross-product for both sides by $R_{d}{}^{-1}$ and $K_{d}$, Equation (3) can be rewritten as:

(5) $$dp=K_{d\;}R_{d}{}^{-1}\left(P_{w}-t_{d}\right),$$

By combining Equations (4) and (5), the pose correlation equation can be rewritten as Equation (6):

(6) $$K_{d\;}R_{d}{}^{-1}\left(P_{w}-t_{d}\right)=K_{rgb\;}\left(R_{rgb}P_{w}+t_{rgb}\right),$$

By making a cross-product for both sides by $K_{rgb}{}^{-1}$, Equation (6) can be rewritten as Equation (7). We derive the relationship between these two different camera models shown in Equation (8):

(7) $$R_{rgb}P_{w}+t_{rgb}=K_{rgb\;}{}^{-1}K_{d\;}R_{d}{}^{-1}P_{w}-K_{rgb\;}{}^{-1}K_{d\;}R_{d}{}^{-1}t_{d},$$

(8) $$R_{rgb}=K_{rgb}{}^{-1}K_{d}R_{d}{}^{-1},\;t_{rgb}=-K_{rgb}{}^{-1}K_{d}R_{d}{}^{-1}t_{d},$$

### 3.3. Initial Camera Tracking

**Figure 2:**
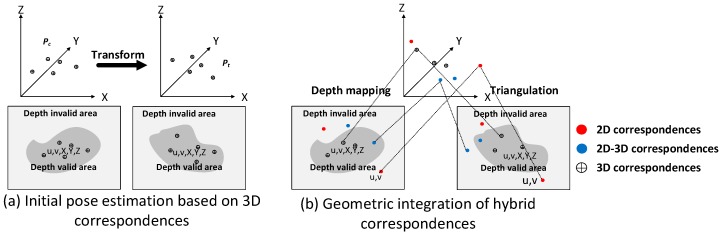
(**a**) Initial pose estimation based on 3D correspondences; (**b**) Geometric integration of hybrid correspondences.

Initial camera poses are essential for pose optimization in a geometric integration model. The minimization problem during poses optimization can be solved and converge faster when the initial poses are more accuracy. In our pipeline, 3D correspondences are first used for initial camera pose estimations. Based on our previous work, which provides a calibration process for depth and RGB cameras [5], 3D coordinates associated 3D correspondence can be obtained from the depth image. To align the current RGB-D frame $F_{c}$ to the target key frame $F_{t}$, two sets of 3D points $P_{c},P_{t}$ can be obtained, associated with 3D correspondences, as shown in Figure 2a. The camera pose tracking is done by iteratively minimizing a robust objective function of the residual errors for sets of 3D measurements $P_{c},P_{t}$. Based on RANSAC and the least square method, the optimal rigid transformation $T^{*}$ between these sets can be calculated as follows:

(9) $$\underset{T}{T^{*}=\mathrm{argmin}}\left(\frac{1}{\left|A\right|}\underset{i\in A}{\sum }w_{i}{\left|T\left(P_{c}^{i}\right)-P_{t}^{i}\right|}^{2}\right),$$

where T is the rigid transformation, comprising a rotation matrix and translation matrix R,t and $T\left(P_{c}^{i}\right)=R*\left(P_{c}^{i}\right)+t$; A contains the associations between feature points in the two frames; and $w_{i}$ is the weight for each point based the theoretical error of depth measurement [5].

In particular, a RANSAC iteration is used for outlier rejection. An initial transformation matrix is calculated with all of the point pairs, including the original point set and the target point set. This initial transformation is applied to the target point set $P_{c}$, after which a new transformed point set $P_{t}$ can be obtained, and the distance between each point pair in transformed point set and the original point set can be calculated and a criterion is set up to robustly filter out the outliers whenever the distance of point pair D is over the Threshold value. RANSAC method is conducted iteratively until no outliers exist. Then, the proper rigid transformation matrix $T^{*}$ between $P_{c}$ and $P_{t}$ is recovered.

### 3.4. Geometric Integration Model

Based on the results of initial camera tracking, the camera motion is refined by a geometric integration process. In addition to the correspondences obtained from the frame-by-frame registration process, we take into account the visual features that appear in multiple frames. By globally minimizing the re-projection error of 3D, 2D-3D and 2D correspondences across all frames, the geometric integration process solves for a set of camera poses and 3D feature points locations.

**Figure 3:**
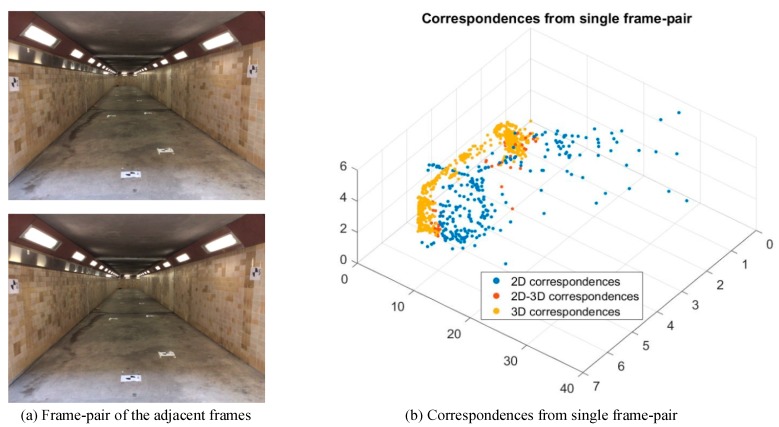
Correspondences identified from single frame pair.

Our system extracts key points from each RGB image and conducts image matching using GPUSIFT. For each key point, the corresponding depth value can be obtained from the depth image. The key points with valid depth value are named as 3D point measurements, otherwise named as 2D point measurements. According to the types of key points, the matched correspondences are categorized as 2D correspondences, 2D-3D correspondences and 3D correspondences, as shown in Figure 2b. 2D correspondences are marked with red dots, which consist of two 2D point measurements. 2D-3D correspondences are marked with blue dots and consist of one 3D point measurement and one 2D point measurement. 3D correspondences marked with a circle-plus sign consist of two 3D point measurements. According to our previous work [5], the 3D coordinates of the points are highly dependent on the accuracy of the depth and the pose of the depth image. In this paper, the initial 3D information of the 3D correspondences are determined using the weighted mean value of the two feature points, while the weight for each point is based on the theoretical error of depth measurement [5]. The initial 3D information of 2D-3D correspondences is calculated from valid depth information, and the 3D coordinates of 2D correspondences are estimated by triangulation based on the initial poses in Section 3.3. Figure 3 shows an example of the correspondences identified from a single frame pair. The blue dot, red dot and yellow dot represent 2D, 2D-3D and 3D correspondences respectively. It should be clear that 2D correspondences are able to provide long-range information through triangulation.

Here, for all of the correspondences, we define a 3D feature points set $D=\{P_{i}^{j}|1\leq i\leq N,1\leq j\leq M,P_{i}^{j}={\left[x_{i}^{j},y_{i}^{j},z_{i}^{j}\right]}^{T}\}$ and a corresponding 2D feature points set $d=\{p_{i}^{j}|1\;\leq i\leq N,1\leq j\leq M,=p_{i}^{j}={\left[u_{i}^{j},v_{i}^{j},1\right]}^{T}\}$, where N means the number of the key frames and *M* means the total number of the correspondences obtained between *i*th frame and the previous adjacent key frame, $X_{i}^{j},Y_{i}^{j},Z_{i}^{j}$ are the associated 3D coordinates in global coordinate system, $u_{i}^{j},v_{i}^{j}$ are the image coordinates of the feature point, $P_{i}^{j}$ and $p_{i}^{j}$ represent the observation of jth feature at ith frame. Upon initialization, all of the features in the sequences are projected into 2D, based on the RGB camera model, $T_{i}\left(P_{i}^{j}\right)$, with the projected 2D points set denoted as ${\tilde{p}}_{i}^{j}={\left[{\tilde{u}}_{i}^{j},{\tilde{v}}_{i}^{j},1\right]}^{T}$. For each correspondence, $E_{i}^{j}$ is defined as the difference between the image reprojection of map point *i* and its actual image measurement:

(10) $$E_{i}^{j}=p_{i}^{j}-{\tilde{p}}_{i}^{j},$$

Define $K_{L}$ as the set of all keyframes and $P_{L}$ as the set of all features in those keyframes $K_{L}$, such that the hybrid geometric integration optimization model can be written as follows:

(11) $$F\left(K_{L},P_{L}\right)=\overset{n}{\underset{i=1}{\sum }}\underset{j\in P_{L}}{\sum }{\left(E_{i}^{j}\right)}^{T}\Omega_{i}^{j}\left(E_{i}^{j}\right),$$

Here, for each feature point, the residual error can be represented by $E_{i}^{j}$, and $\Omega_{i}^{j}$ is the information matrix of the feature point and is used for weight representation. Depth uncertainty determines the weight of each correspondence. For the 3D correspondence and 2D-3D correspondence, the first two entries on the diagonal of $\Omega_{i}^{j}$ are given, based on the depth error model proposed in our previous calibration work [5]. For the 2D correspondences, the weights are constant values and smaller. 

In our work, all image measurements are utilised to compute optimal pose updates for the keyframe set. During the bundle adjustment processing, the poses of all keyframes and the 3D information of the feature points are optimized, except for the original keyframes that are fixed to eliminate the gauge freedom. Thus, the geometric integration system means solving the following minimizations problem:

(12) $$\left\{P_{i}^{j},T_{i}|i\in K_{L},j\in P_{L}\right\}\underset{T_{i},P_{i}^{j}}{=\mathrm{argmin}}F\left(K_{L},P_{L}\right)$$

This can be solved by iterations of reweighted nonlinear least squares. One fundamental requirement to do this efficiently is to differentiate measurement errors, to obtain their Jacobians, with respect to those parameters that need to be estimated. In geometric integration adjustment, the derivatives of $E_{i}^{j}$ with respect to $T_{i}\left(P_{i}^{j}\right)$ and the map point position $P_{i}^{j}$ are required. In our system, quaternions are used to provide a convenient mathematical notation for representing camera orientations. Thus, for a map point j in the frame i, we can compute the Jacobian matrix of $E_{i}^{j}$ with respect to the camera pose $T_{i}\left(P_{i}^{j}\right)$ using the chain rule, as:

(13) $$J_{T_{i}\left(P_{i}^{j}\right)\;}=\frac{\partial \left(E_{i}^{j}\right)}{\partial \left(T_{i}\right)}=\frac{\partial \left(E_{i}^{j}\right)}{\partial \left(c\right)}{|}_{c=T_{i}\left(P_{i}^{j}\right)}\cdot \frac{\partial \left(T_{i}\left(P_{i}^{j}\right)\right)}{\partial \left(T_{i}\right)},$$

The first term of the above matrix product is the Jacobian of the camera projection function, and the last term is:

(14) $$\frac{\partial \left(T_{i}\left(P_{i}^{j}\right)\right)}{\partial \left(T_{i}\right)}=[\begin{array}{ccccccc} \frac{\partial \left(E_{i}^{j}{}_{x}\right)}{\partial q_{0}} & \frac{\partial \left(E_{i}^{j}{}_{x}\right)}{\partial q_{1}} & \frac{\partial \left(E_{i}^{j}{}_{x}\right)}{\partial q_{2}} & \frac{\partial \left(E_{i}^{j}{}_{x}\right)}{\partial q_{3}} & \frac{\partial \left(E_{i}^{j}{}_{x}\right)}{\partial t_{x}} & \frac{\partial \left(E_{i}^{j}{}_{x}\right)}{\partial t_{y}} & \frac{\partial \left(E_{i}^{j}{}_{x}\right)}{\partial t_{z}}\\ \frac{\partial \left(E_{i}^{j}{}_{x}\right)}{\partial q_{0}} & \frac{\partial \left(E_{i}^{j}{}_{x}\right)}{\partial q_{1}} & \frac{\partial \left(E_{i}^{j}{}_{x}\right)}{\partial q_{2}} & \frac{\partial \left(E_{i}^{j}{}_{x}\right)}{\partial q_{3}} & \frac{\partial \left(E_{i}^{j}{}_{x}\right)}{\partial t_{x}} & \frac{\partial \left(E_{i}^{j}{}_{x}\right)}{\partial t_{y}} & \frac{\partial \left(E_{i}^{j}{}_{x}\right)}{\partial t_{z}} \end{array}],$$

Similarly, the Jacobian matrix of $E_{i}^{j}$ with respect to the map-point position $P_{i}^{j}$ can be expressed in a consistent way:

(15) $$J_{T_{i}\left(P_{i}^{j}\right)\;}=\frac{\partial \left(E_{i}^{j}\right)}{\partial \left(P_{i}^{j}\right)}=\frac{\partial \left(E_{i}^{j}\right)}{\partial \left(c\right)}{|}_{c=T_{j}\left(P_{i}^{j}\right)}\cdot \left[\begin{array}{ccc} \frac{\partial \left(E_{i}^{j}{}_{x}\right)}{\partial X} & \frac{\partial \left(E_{i}^{j}{}_{x}\right)}{\partial Y} & \frac{\partial \left(E_{i}^{j}{}_{x}\right)}{\partial Z}\\ \frac{\partial \left(E_{i}^{j}{}_{y}\right)}{\partial X} & \frac{\partial \left(E_{i}^{j}{}_{y}\right)}{\partial Y} & \frac{\partial \left(E_{i}^{j}{}_{y}\right)}{\partial Z} \end{array}\right],$$

### 3.5. Loop Closure Detection and Global Optimization

Successive frame alignment results in accumulating drift error over time. This is more significant when there is a long camera trajectory. Loop closure detection is a solution to this problem. The simplest method is to implement a linear search of all the existing key frames and to align the current frame with the target key frame. However, the computational cost increases rapidly as the number of key frames grows. Here, we employ a combination of techniques to enhance the efficiency and accuracy of loop closure detection, which contains movement-based and bag-of-word-based. 

**Figure 4:**
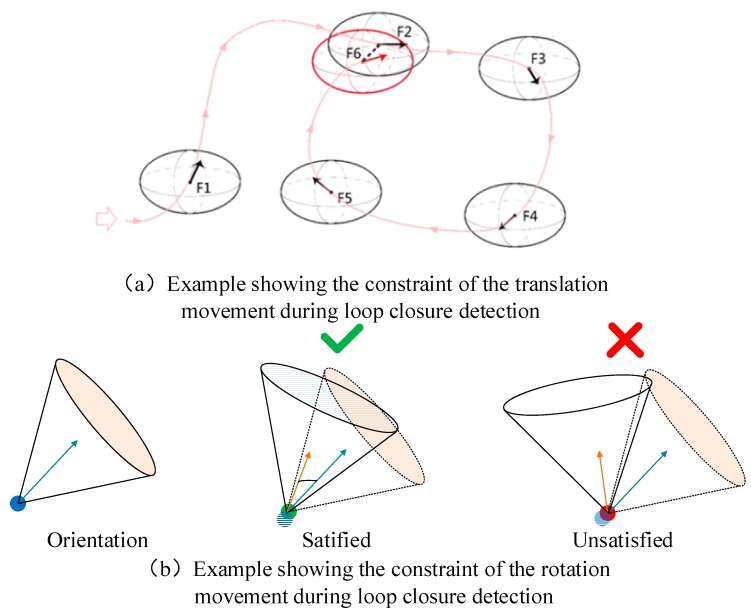
Examples showing the usage of movement metric for loop closure detection.

As the image stream is continuous, loop closure is most likely to be found when two frames have a short movement distance. Therefore, for each frame, we utilize a movement metric sensitive to both rotation and translation which indicates when to add a new frame to the place recognition. We first define an ellipsoid region for the current frame in Equation (16), in which the origin $\left(tx_{i},ty_{i},tz_{i}\right)$ is the position of the current frame and a,b,c correspond to the semi-major axis and semi-minor axis of the ellipse. If this movement metric of translation, $m_{t}$ is below 1, it means the compared frame located in the ellipsoid. The rotation movement metric is then quantized by Equation (17), it calculates the angle movement of the rotation between the current frame and the target frame. The loop closure candidate is recognized when both $m_{r}$ goes below a specified angle $\theta_{r}$ threshold and $m_{t}$ goes below 1, as Figure 4 shown. Empirically, we found $a=0.5,\;b=0.5,\;c=0.3,\;\theta_{r}=15{}^{\circ}$ provides good performance:

(16) $$m_{t}=\frac{{\left(tx_{j}-tx_{i}\right)}^{2}}{a^{2}}+\frac{{\left(ty_{j}-ty_{i}\right)}^{2}}{b^{2}}+\frac{{\left(tz_{j}-tz_{i}\right)}^{2}}{c^{2}}$$

(17) $$m_{r}=\cos{<\underset{R_{i}}{\to },\underset{R_{j}}{\to }>}^{-1}=\cos^{-1}\frac{\underset{R_{i}}{\to }\cdot \underset{R_{j}}{\to }}{\left|\underset{R_{i}}{\to }\right|\cdot \left|\underset{R_{j}}{\to }\right|}=\cos^{-1}\frac{r_{i}^{x}r_{j}^{x}+r_{i}^{y}r_{j}^{y}+r_{i}^{z}r_{j}^{z}}{\sqrt{r_{i}^{x}{}^{2}+r_{i}^{y}{}^{2}+r_{i}^{z}{}^{2}}\cdot \sqrt{r_{j}^{x}{}^{2}+r_{j}^{y}{}^{2}+r_{j}^{z}{}^{2}}}$$

Upon receiving a new loop closure candidate, we use SiftGPU descriptors with the bag-of-words-based DBoW loop detector for place recognition [34]. The existing bag-of-words descriptor database is queried. If a match is found, the SIFT features and the matches would be used for rigid transformation computation. If the best rigid transformation between two key frames can be recovered by the pose estimation methods mentioned in Section 3.2.2, the loop closure is recognized and the corresponding adjacent edge is added to the pose graph.

In particular, when modeling an indoor environment, we always end with the same scene as in the beginning frames, thereby making a circle. The beginning frames and the ending frame may contain relationships. Therefore, we also search for a loop closure between the beginning frames and the ending frames.

Our strategy of global optimization is to represent the loop closure constraint with a vertex-edge pose graph. Vertexes contain the initial pose of the key frames and edges represent the rigid transformation between the key frames obtained from the frame registration and loop closure detection processes. Because there is significant inconsistency among these geometric constraints and the error is distributed over the edges in the graph, the inconsistency can be corrected by solving a nonlinear least-squares optimization problem.

## 4. Experimental Analysis

### 4.1. TUM Datasets Experiments

**Figure 5:**
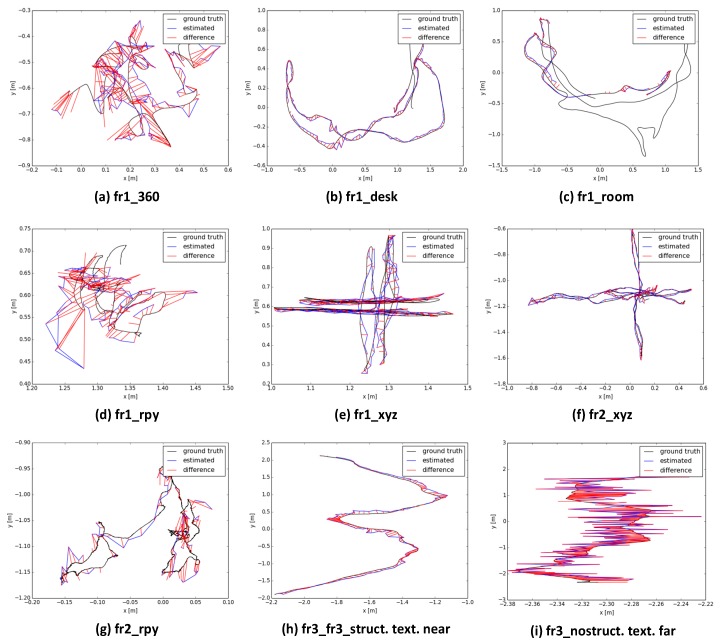
Comparison of estimated and ground truth trajectories.

We first evaluated the hybrid SLAM method using the publicly available RGB-D benchmark dataset from TUM datasets [35], which contain ground truth information for camera poses organized in time-series, are used to assess the accuracy of the camera trajectory. The results are compared with some state-of-the-art SLAM methods. Nine sequences are processed and the quantitative results are shown in Table 1 and Figure 5. The median (max) absolute trajectory error (ATE) is calculated by the automated evaluation tool provided by [35]. It calculates the RMSE of the Euclidean distances between the camera trajectory and the timestamp associated ground truth, and it is used to evaluate the procedure’s accuracy. 

As shown in Table 1, the proposed SLAM system achieves consistent results in all of the RGB-D sequences. The median of the absolute trajectory errors is within 6 cm. Figure 5 shows the estimated and true camera trajectories and the difference between them. In our system, the original image streams are sampled after the key frames have been detected and only the poses of the key frames are visualized. Therefore, the estimated trajectories shown with the blue line are not as dense as the true trajectory, which is shown with a black line.

**Figure 6:**
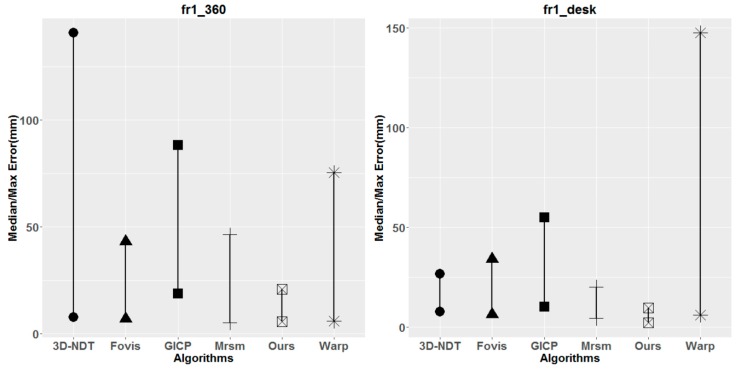

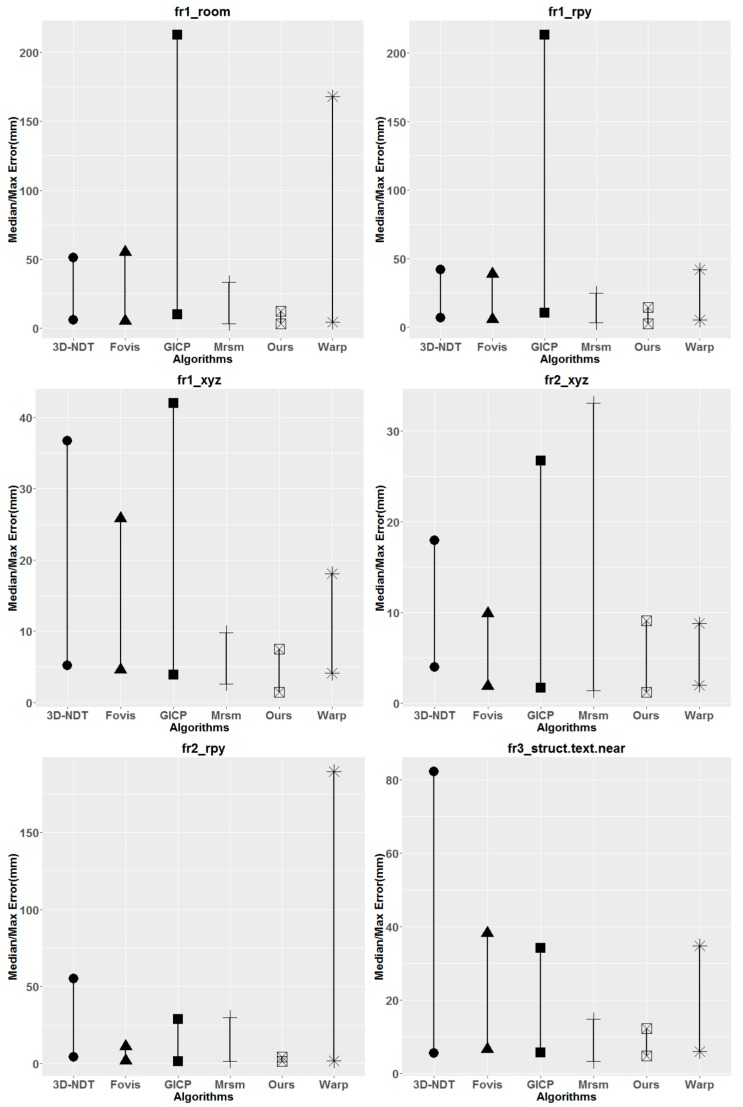

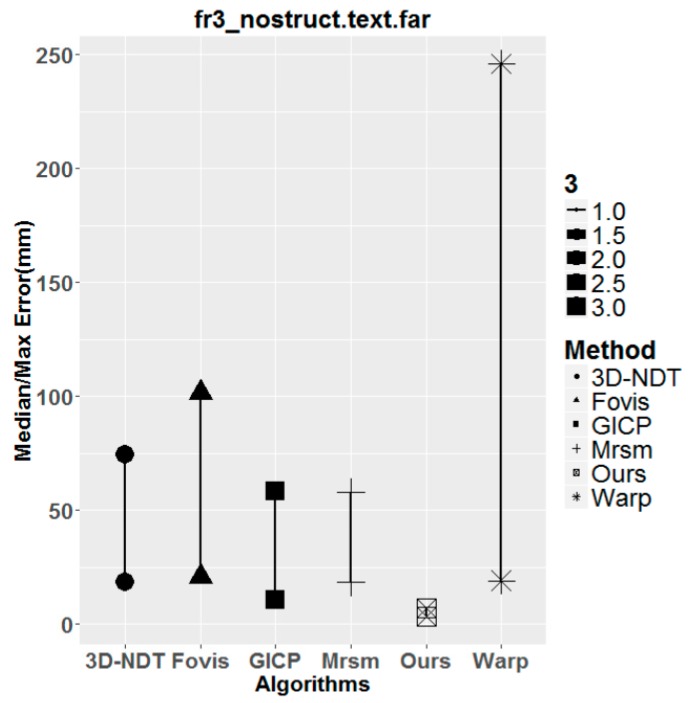
Comparisons of the median/max absolute trajectory error (ATE) in mm for incremental registration on RGB-D sequences from the Freiburg benchmark dataset. In each plot, different vertexes represent different methods. The higher vertexes represent the max ATE and the lower vertexes are the median ATE.

To demonstrate the performance of our SLAM pipeline, we compare the experimental results of our system with those of five other SLAM methods, i.e., Mrsm [17], Warp [22], GICP [36], 3D-NDT [37], Fovis [21], RGB-D [2], dense SLAM [18] and VP [15]. Specifically, only some sets of the results can be obtained from RGB-D, dense SLAM and VP method. All of the results are presented in Table 1 and plotted in Figure 6. As shown in Table 1, it is easy to see that our method outperforms the others in most sequences. In the sequences of the fr1_room, fr1_rpy, fr2_xyz and fr3_nostruct.text.far sequences, our system achieves the best results in median value of ATE. For the other sequences, the proposed hybrid SLAM method achieve secondary accuracy in median value of ATE. For the maximum value of ATE, the proposed hybrid SLAM system achieve higher accuracy than others in most cases. The results indicate that our SLAM system is able to provide better stability than the other methods and higher accuracy in most cases.

For the fr2_rpy sequence, because the scene provides both geometric and visual features, both the point feature-based method and structure-based method achieve high accuracy, within 5 cm of the median ATE. In the fr1_360 and fr3_structure.text.near sequences, they contain abundant geometric structures. As Mrsm is structure-based, it performs better than the other methods, which indicates that the Mrsm algorithm may complement our visual-based SLAM system in scenes with an abundance of structures. 

In this paper, all experiments were carried out on an Intel Core i7-4600U CPU with 8 GB of memory. Graph optimization is started after all frames have been processed. The runtime results presented in Table 2 were generated using SIFTGPU and FLANN matching. During SLAM processing, feature detection and matching is costly to compute. On average, our system achieve 0.37 s per frame. For the global optimization, the optimization of small pose graph is fast enough to be done in real time. The optimization time increases with the number of the key frames and loop closure. 

### 4.2. Unidirectional Tracking Experiments

#### 4.2.1. Datasets Collection

**Figure 7:**
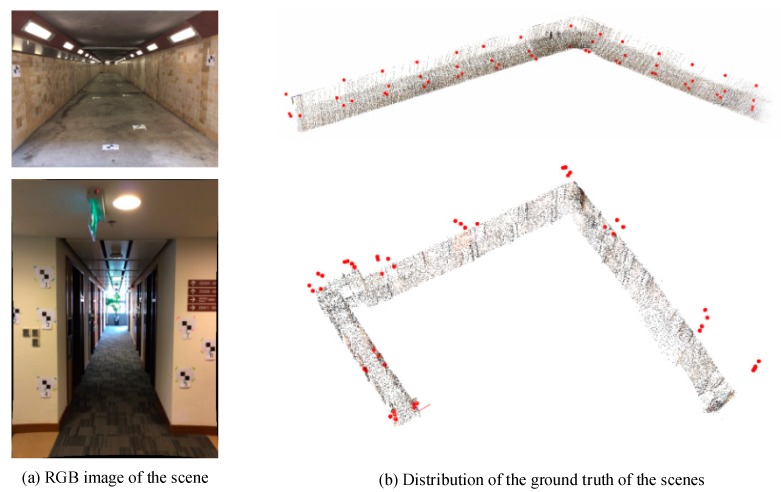
Ground truth obtained from total station.

Experiments were carried out to evaluate the effectiveness of the proposed geometric integration method. We evaluated mapping accuracy with four sets of RGB-D sequences recorded in a tunnel and a corridor with a handheld Structure Sensor device. Evaluating the accuracy of the SLAM methods requires ground truth data. As shown in Figure 7, 54 and 46 targets are evenly distributed along the tunnel and corridor, and measured by total station, which serves as ground truth for accuracy evaluation and comparison.

**Figure 8:**
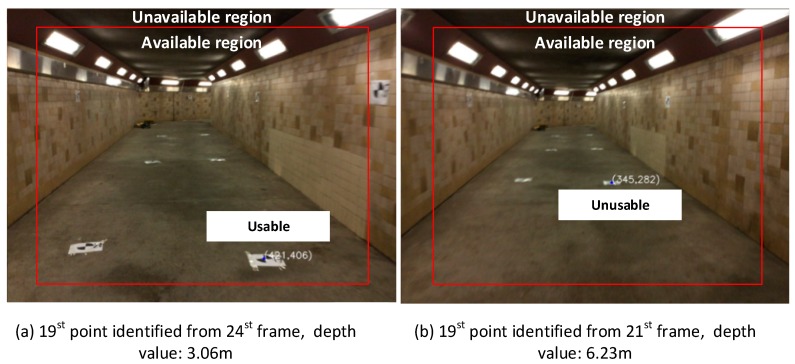
Strategy of checkpoints determination.

For checkpoint identifications, with targets numerically marked, we automatically extracted the corresponding points from the RGB-D sequences and then obtained their 3D location by mapping them to the depth image. For each RGB-D sequence, four point-cloud sets are obtained from different SLAM pipelines and used for checking purposes. Figure 8 shows the strategy for checkpoint determination.

For each checkpoint, we use three conditions to obtain the optimal result: (1) to avoid the impact from depth uncertainty, only the points with a depth value less than 3.5 m are considered; (2) as each checkpoint can be identified from multiple frames, only the point with minimal depth value is considered; and (3) the identified point should be located in the available region on the image, which is bounded by a 30 × 20 pixels patch. As shown in Figure 8, the 19th point can be identified from 24th and 21st frames, the depth values of which are 3.06 m and 6.23 m, respectively. According to our checkpoint determination strategy, only the point obtained from the 24th frame is used as the checkpoint.

To quantify our measurement results, we show four sets of experiments in which we compare four outputs: (i) SLAM results with the mapping system of Structure Sensor, which we call Structure Sensor SLAM; (ii) SLAM without the use of 2D-3D and 2D correspondences, which we call 3D SLAM; (iii) the open-source RGB-D SLAM system, ORB SLAM; and (iv) Hybrid SLAM, which uses all of the correspondences. Two kinds of error metrics are used. The first is the absolute mapping accuracy, which is presented by the RMSE of the checkpoints relative to the ground truth. RMSE residual error is defined as the root mean square of residual distances between the checkpoints and the ground truth. It should be noted that the initial positions of the ground truth and SLAM system are the same. The second is the ratio of the mapping errors to the camera trajectory length, which can be used to normalize mapping accuracy and to evaluate the velocity of drift accumulation. 

#### 4.2.2. Experimental Results from Tunnel Scenes

**Figure 9:**
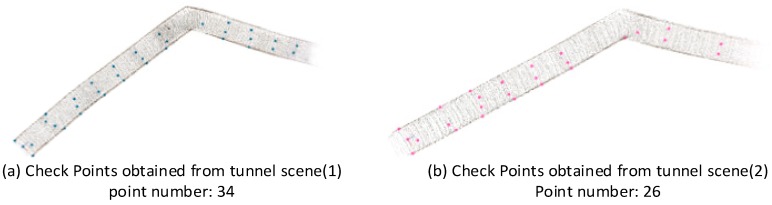
Tunnel scenes, marked with the checkpoints, (**a**) checkpoints obtained from tunnel scene (1); (**b**) checkpoints obtained from tunnel scene (2).

It should be noted that not all of the target points in the tunnel experiments can be identified because of the view angle of the camera and an overexposure problem. To check the results of the geometric integration, 34 checkpoints in tunnel scene (1) and 26 checkpoints in tunnel scene (2) were identified according to the checkpoint determination strategy, as shown in Figure 9. 3D coordinates in the object space were derived from the SLAM results, of which the camera pose of each key frame was optimized during SLAM processing. Thus, four sets of checkpoints can be obtained from Structure Sensor SLAM, 3D SLAM, ORB SLAM and hybrid SLAM solutions.

**Figure 10:**
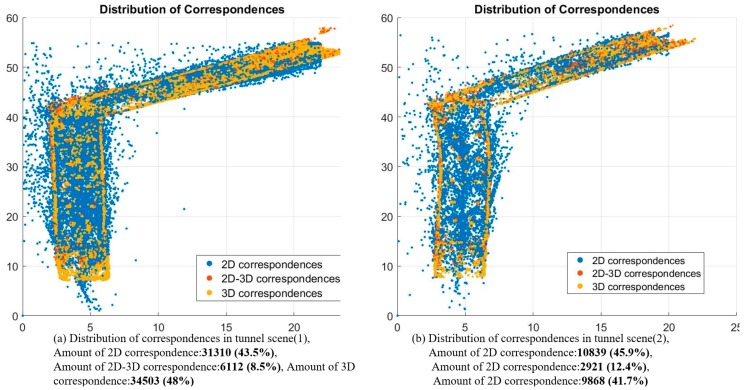
Distribution of correspondences of tunnel scenes.

The distribution of different types of correspondences are first showns in Figure 10. The blue, red and yellow dots represent the 2D, 2D-3D and 3D correspondences, respectively. The total percentage of 2D-3D and 2D correspondences is about 52% in tunnel scene (1) and 57% in tunnel scene (2), respectively, and the percentage of 3D correspondences are below 50%. This demonstrates that correspondences without valid depth values occupy a large proportion of the available information. The results also indicate the significant long-range constraints during camera tracking and graph optimization.

**Figure 11:**
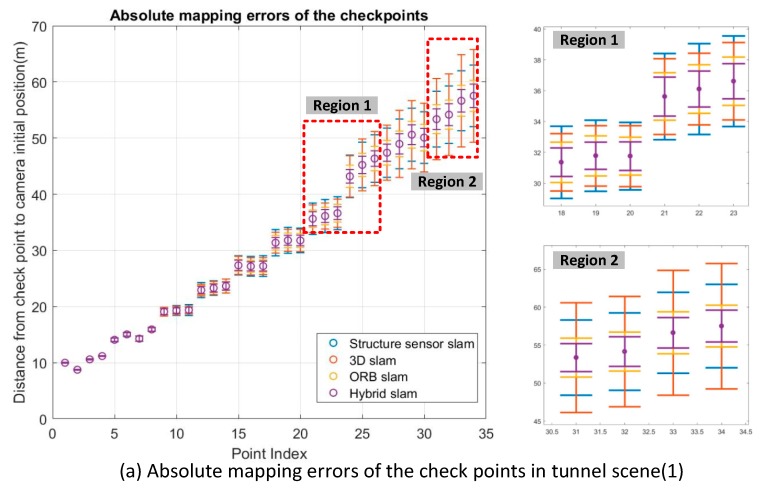

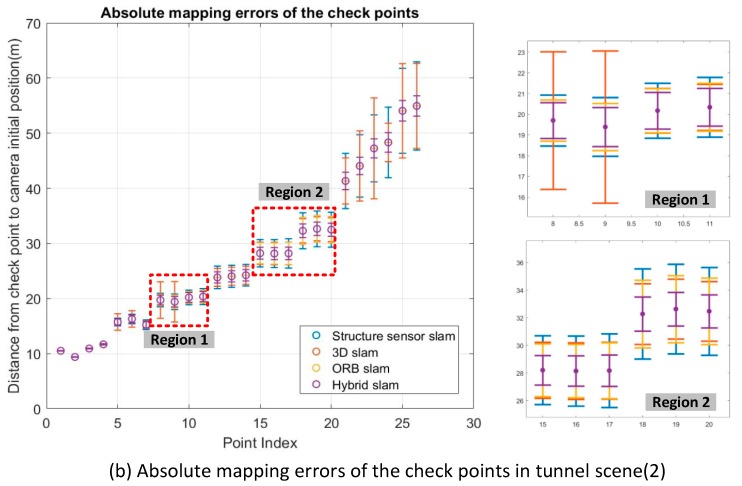
Absolute mapping errors of the checkpoints along with camera trajectories.

To evaluate the absolute mapping accuracy of the different SLAM methods, a rigid transformation was first applied to these four sets of checkpoints using four control points, such that the initial positions of ground truth and the SLAM system were the same. The mapping accuracies of these four methods, Structure Sensor SLAM, 3D SLAM, ORB SLAM and hybrid SLAM, were compared using the 34 and 26 checkpoints previously mentioned. The discrepancies were calculated by comparing the checkpoint sets derived from the four SLAM methods against the ground truth. If there was no bias in the camera poses after SLAM, the positions of the checkpoints should be the same as the ground truth. However, Figure 11 indicates that this was not the case. In Figure 11, the X axis shows that the point index increased with the camera trajectories and the Y axis denotes the absolute distances of the checkpoints to the initial camera position. The discrepancies for each checkpoint with the different SLAM methods are presented by an error bar with different colors. As expected, the mapping errors grow linearly as the point index increases in both tunnel scenes (1) and (2). We highlight two error bar segments that correspond to the regions marked by red rectangle in Figure 11 to clarify the tracking results of different SLAM systems. This illustrates that the absolute mapping errors from hybrid SLAM are much smaller than those in all other cases. Table 3 lists the detailed statistics of absolute mapping errors and the ratio of the mapping errors to trajectory lengths using different SLAM methods in both tunnel scenes. As the ORB SLAM failed to track in the middle of tunnel scene (2), we calculate the ratio of the error with the corresponding trajectory length, which is 26 m. From Table 3 the following can be noted. The Structure Sensor SLAM method produces a mapping accuracy of 2.96 m in tunnel scene (1) and 3.56 m in tunnel scene (2). The 3D SLAM method produces a mapping accuracy of 3.73 m in tunnel scene (1) and 3.68 m in tunnel scene (2). ORB SLAM achieved a mapping accuracy 1.55 m in tunnel scene (1) and 1.17 m (with trajectory length of 26 m) in tunnel scene (2). The Structure Sensor SLAM and 3D SLAM generated similar results, as they used the same feature detector and only use 3D correspondences for camera tracking. The hybrid SLAM method outperforms all of other approaches in both tunnel scenes (1) and (2).

Based on the hybrid SLAM method, the ratios of mapping error to the trajectory length are about 2% in both tunnel scenes (1) and (2). The RMSE of the discrepancies decreased from 3.73 to 1.14 m in tunnel scene (1) and from 3.68 to 1.13 m in tunnel scene (2) after hybrid SLAM, indicating relative improvements of 4.6% in tunnel scene (1) and 4.1% in tunnel scene (2).

#### 4.2.3. Experimental Results from Corridor Scenes

**Figure 12:**
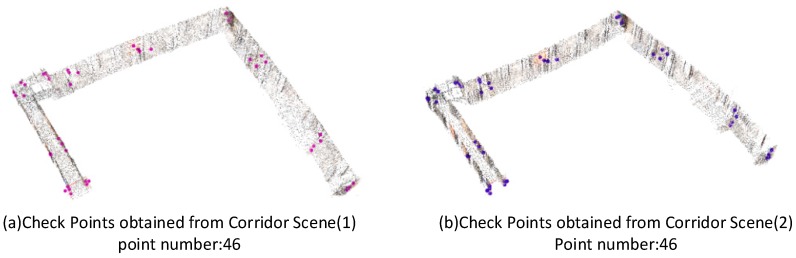
Corridor scenes, marked with the checkpoints, (**a**) checkpoints obtained from corridor scene (1); (**b**) checkpoints obtained from corridor scene (2).

Forty-six checkpoints were identified manually to confirm the results of the geometric integration in corridor scenes. The distributions of the checkpoints are marked in corridor scenes (1) and (2), as shown in Figure 12. Corridor scenes, marked with the checkpoints, (a) checkpoints obtained from corridor scene (1); (b) checkpoints obtained from corridor scene (2).

**Figure 13:**
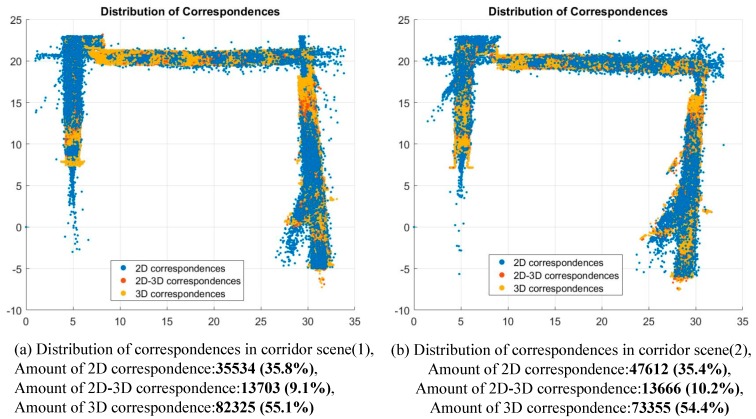
Distribution of correspondences of corridor scenes.

The distribution of different types of correspondences, including 2D correspondences, 2D-3D correspondences, and 3D correspondences, are shown in Figure 13. Distribution of correspondences of corridor scenes. The total percentage of 2D-3D correspondences and 2D correspondence is about 45% in both corridor scenes (1) and (2), which is much smaller than that in the tunnel scenes due to fewer valid depth measurements in each frame.

**Figure 14:**
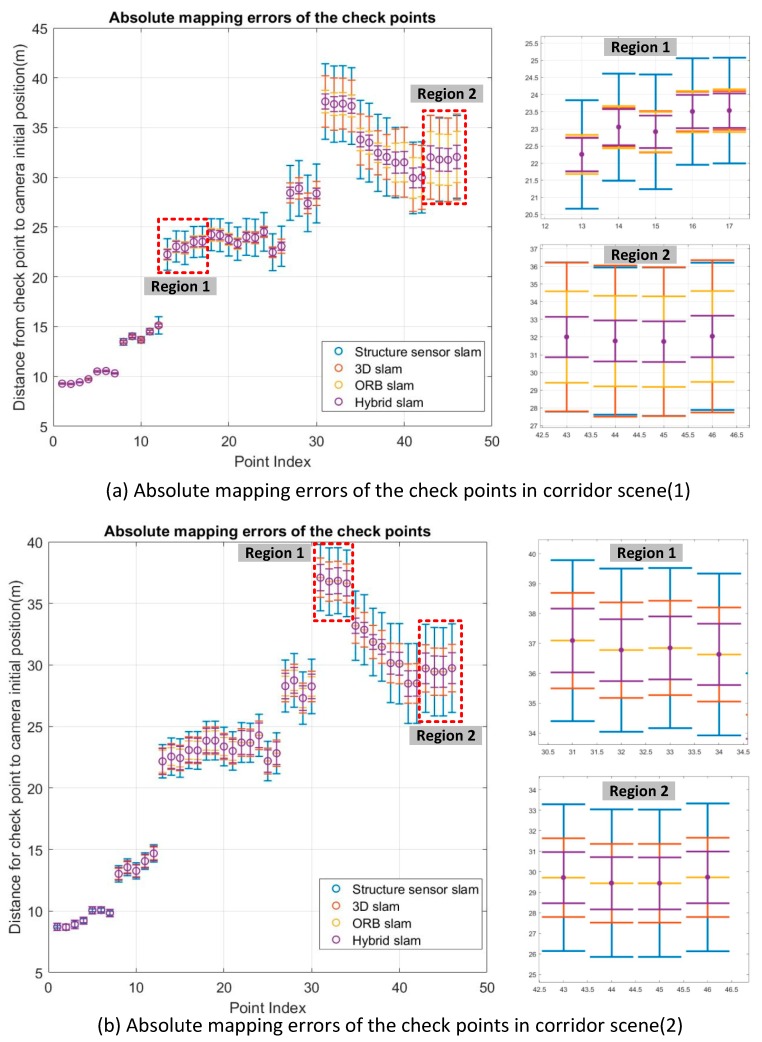
Absolute mapping errors of the checkpoints along with camera trajectories.

The checkpoints set and the ground truth of the corridor scenes were initially aligned by control points identified from the first RGB-D frame. The performances of the geometric integration approaches were evaluated by comparing the ground coordinates derived from the three methods with the ground truth data. The discrepancies of each checkpoint from different SLAM methods are presented by error bars with different colors, as shown in Figure 14. Absolute mapping errors of the checkpoints along with camera trajectories. Similarly, the drift errors are accumulated as the point index increases in both corridor scenes (1) and (2). From the error bar segments corresponding to the part of error bars marked in red rectangle regions in Figure 14. Absolute mapping errors of the checkpoints along with camera trajectories, the hybrid SLAM method achieved a much higher mapping accuracy than the others.

Table 4 lists the statistics of the absolute mapping errors and the ratio of the mapping error to trajectory length using each SLAM method in the corridor scenes. Due to the pure rotation problem, ORB SLAM failed to track in the middle of corridor scene (2) and the trajectory length was about 39.2 m. As expected, the hybrid SLAM method outperformed the other approaches in both corridor scenes (1) and (2), again verifying its effectiveness. The ratios of mapping error to trajectory lengths are 0.98% and 1.3% in corridor scenes (1) and (2), respectively. The RMSE of the discrepancies decreased from 2.13 to 0.63 m in corridor scene (1) and from 2.62 to 0.87 m in corridor scene (2), after geometric integration of hybrid correspondences. The relative improvements of hybrid SLAM are 2.3% in corridor scene (1) and 2.8% in corridor scene (2). The accuracy improvements between 3D SLAM and hybrid SLAM are more significant in the tunnel scenes (over 4%) than that in corridors scenes (under 3%), probably due to the higher ratio of the 2D-3D and 2D correspondences in tunnel scenes. 

## 5. Discussion

The objective of this study was to explore the effectiveness of the use of hybrid correspondences in RGB-D SLAM, especially in no-loop-closure environments. As the use of region without valid depth has rarely been investigated in existing RGB-D SLAM systems. We attempt to integrate the 2D correspondences, 2D-3D correspondences and 3D correspondences according to a rigid mathematical method for trajectories optimization by globally minimizing the re-projection error of the hybrid correspondences across all key-frames. Experiments involving nine sets of TUM datasets and four sets of RGB-D sequences recorded with structure sensor device were carried out to evaluate the effectiveness of the proposed geometric integration method. 

The results indicates that the hybrid SLAM method achieves higher mapping accuracy and outperforms all of other approaches in all cases. Based on the hybrid SLAM method, the ratios of mapping error to the trajectory length are about 2% in both tunnel scenes (1) and (2). The RMSE of the discrepancies decreased from 3.73 to 1.14 m in tunnel scene (1) and from 3.68 to 1.13 m in tunnel scene (2) after hybrid SLAM, indicating relative improvements of 4.6% in tunnel scene (1) and 4.1% in tunnel scene (2). As expected, the hybrid SLAM method outperformed the other approaches in both corridor scenes (1) and (2), again verifying its effectiveness. The consistent results can be achieved because the hybrid SLAM method incorporate the new long-range constraints from 2D and 2D-3D correspondences. In horizontal comparison, the accuracy improvements between 3D SLAM and hybrid SLAM are more significant in the tunnel scenes (over 4%) than that in corridors scenes (under 3%). The difference probably caused by the distribution and the ratio of the 2D and 2D-3D correspondences. Higher ratio of 2D and 2D-3D correspondences tend to provide more constraints during tracking or global optimization. 

Even though the results in this article exhibit a positive contribution of the proposed geometric integration model, there are still several associated aspects that need to be studied with more detailed investigations. The contribution of each correspondence during global optimization should be modeled carefully according to the degree of reliability of depth, instead of using a constant weight. Moreover, the proposed geometric integration SLAM method mainly concentrates on the multi-dimension visual features of RGB sequences regardless of the geometric features from depth images. We will investigate the combination of visual and geometric features for RGB-D SLAM in the not-so-distant future.

## 6. Conclusions

This paper presents a geometric integration model to integrate 2D, 2D-3D and 3D correspondences and improve mapping accuracy during RGB-D unidirectional tracking. Through theoretical analysis and experimental validation, the following conclusions can be drawn.

(1) The proposed method provides new opportunities to use 2D correspondences to provide long-range constraints in the camera trajectory optimization process, which is able to effectively decrease drift error and improve the scene mapping accuracies during RGB-D unidirectional tracking.
(2) For corridor and tunnel scenes, the mapping accuracies obtained after geometric integration with hybrid correspondences were significantly improved. The RMSE of the discrepancies decreased from 3.73 to 1.14 m in tunnel scene (1) and from 3.68 to 1.13 m in tunnel scene (2), from 2.13 to 0.63 m in corridor scene (1) and from 2.62 to 0.87 m in corridor scene (2) after hybrid SLAM. The hybrid SLAM system outperforms the traditional 3D SLAM, Structure Sensor SLAM and ORB SLAM systems in all cases.
(3) The performance benefit from the hybrid SLAM method was even greater for the tunnel scenes than the corridor scenes due to the higher ratio of 2D-3D and 2D correspondences in the tunnel scene. The ratio of different correspondences may have effects on the performance of hybrid SLAM approach.

The geometric integration model discussed here enables the integration of multi-type correspondences for RGB-D camera tracking. This encourages a more accurate derivation of RGB-D camera tracking, permitting a full comparative and synergistic use of diverse feature types. The geometric integration strategy can also be used in other similar systems such as the integrated processing of visual images and terrestrial laser scanning data. The next step in this research will to be improve the hybrid SLAM method by combining the geometric features, such as 3D points, 3D line and 3D plane, which would make the method more robust when mapping in less textured regions or dark spaces. 

## Figures and Tables

**Table 1:** Comparisons of the median (max) absolute trajectory error (ATE; in mm) for incremental registration of RGB-D sequences of the Freiburg Benchmark Dataset. Best results in bold.

	Method	fr1_360	fr1_desk	fr1_room	fr1_rpy	fr1_xyz	fr2_xyz	fr2_rpy	fr3_struct. text.near	fr3_nostruct. text.far
MedianATE	Hybrid SLAM	5.7	2.2	**3.2**	**2.2**	1.4	**1.2**	1.4	4.8	**3.2**
	Mrsm	**5.1**	4.4	3.5	3	2.6	1.4	1.6	**3.2**	18.5
	Warp	5.9	6.2	4.6	5.1	4.1	2	1.7	5.9	19.2
	GICP	18.8	10.2	10.2	10.4	3.9	1.7	**1.3**	5.6	10.9
	3D-NDT	7.8	7.8	6.1	6.8	5.2	4	4.2	5.5	18.6
	Fovis	7.1	6.3	5.4	5.4	4.6	1.9	1.7	6.5	20.8
	RGB-D	/	2.3	8.4	2.6	**1.3**	2.0	/	/	/
	Dense SLAM	/	3.7	7.5	2.8	1.7	2.9	/	/	/
	VP	/	**1.8**	14.4	/	/	/	/	/	/
MAX ATE	Hybrid SLAM	**21**	9.7	**12**	**14**	**7.5**	9.1	**4.4**	**12.2**	**7**
	Mrsm	46.4	20.1	33.3	24.8	9.8	33.1	29.9	14.7	57.9
	Warp	75.4	147	168	41.8	18.1	**8.8**	190	34.8	246
	GICP	88.3	54.9	213	213	42	26.8	28.7	34.2	58.6
	3D-NDT	141	26.6	51.2	41.9	36.7	18	55.1	82.3	74.6
	Fovis	43.1	34.2	55.1	38.7	25.8	9.9	11	38.2	101.5
	VP	/	**6.6**	33.9	/	/	/	/	/	/

**Table 2:** Running time of the TUM RGB-D sequences.

Sequences	Frames	Total Runtime	g2oRuntime
fr1_360	756	151 s	0.56 s
fr1_desk	613	165 s	1.44 s
fr1_room	1362	418 s	1.66 s
fr1_rpy	723	225 s	9.89 s
fr2_xyz	3669	1462 s	21.34 s
fr2_rpy	3290	1387 s	18.78 s
fr3_struct.text.Near	938	451 s	4.21 s
fr3_nostruct.text.Far	465	123 s	1.13 s

**Table 3:** Absolute mapping accuracy of tunnel scenes using different SLAM methods.

Datasets	Number of Checkpoints	Trajectory Length (m)	RMSE of the Discrepancies from the Checkpoints with Different Method (m)				Relative Improvements (%)
			Structure Sensor SLAM	3D SLAM	ORB SLAM	Hybrid SLAM	
Tunnel scene (1)	34	56.5	2.96	3.73	1.55	1.14	4.6
			(−5.2%)	(−6.6%)	(−2.7%)	(−2%)	
Tunnel scene (2)	26	56.4 (26 in ORB SLAM)	3.56	3.68	1.17	1.13	4.1
			(−6.3%)	(−6.5%)	(−4.6%)	(−2%)	

**Table 4:** Absolute mapping accuracy of corridor scenes using different SLAM method.

Datasets	Number of Checkpoints	Trajectory Length (m)	RMSE of the Discrepancies from the Checkpoints with Different Method (m)				Relative Improvement (%)
			Structure Sensor SLAM	3D SLAM	ORB SLAM	Hybrid SLAM	
Corridor scene (1)	46	64.1	2.13	1.96	1.12	0.63	2.3
			(−3.3%)	(−3.1%)	(−1.8%)	(−0.98%)	
Corridor scene (2)	46	63.7 (39.2 in ORB SLAM)	2.62	1.21	0.62	0.87	2.8
			(−4.1%)	(−1.9%)	(−1.6%)	(−1.3%)	
